# Predicting response to physiotherapy treatment for musculoskeletal shoulder pain: protocol for a longitudinal cohort study

**DOI:** 10.1186/1471-2474-14-192

**Published:** 2013-06-21

**Authors:** Rachel Chester, Lee Shepstone, Jeremy S Lewis, Christina Jerosch-Herold

**Affiliations:** 1 School of Allied Health Professions, Faculty of Medicine and Health Sciences, University of East Anglia, Norwich, Norfolk NR4 7TJ, UK; 2 Physiotherapy Department, Norfolk and Norwich University Hospital, Norwich, Norfolk NR4 7UY, UK; 3 Norwich Medical School, Faculty of Medicine and Health Sciences, University of East Anglia, Norwich, Norfolk NR4 7TJ, UK; 4 Department of Allied Health Professions, School of Health and Social Work, University of Hertfordshire, College Lane, Hatfield AL10 9AB, UK; 5 Musculoskeletal Department, Health at the Stowe, Central London Community Healthcare, 260 Harrow Road, Greater London W2 5ES, UK

**Keywords:** Physical therapy, Shoulder, Shoulder pain, Musculoskeletal, Predict, Prognosis

## Abstract

**Background:**

Shoulder pain affects all ages, with a lifetime prevalence of one in three. The most effective treatment is not known. Physiotherapy is often recommended as the first choice of treatment. At present, it is not possible to identify, from the initial physiotherapy assessment, which factors predict the outcome of physiotherapy for patients with shoulder pain. The primary objective of this study is to identify which patient characteristics and baseline measures, typically assessed at the first physiotherapy appointment, are related to the functional outcome of shoulder pain 6 weeks and 6 months after starting physiotherapy treatment.

**Methods/Design:**

Participants with musculoskeletal shoulder pain of any duration will be recruited from participating physiotherapy departments. For this longitudinal cohort study, the participants care pathway, including physiotherapy treatment will be therapist determined.

Potential prognostic variables will be collected from participants during their first physiotherapy appointment and will include demographic details, lifestyle, psychosocial factors, shoulder symptoms, general health, clinical examination, activity limitations and participation restrictions.

Outcome measures (Shoulder Pain and Disability Index, Quick Disability of the Arm, Shoulder and Hand, and Global Impression of Change) will be collected by postal self-report questionnaires 6 weeks and 6 months after commencing physiotherapy.

Details of attendance and treatment will be collected by the treating physiotherapist. Participants will be asked to complete an exercise dairy.

An initial exploratory analysis will assess the relationship between potential prognostic factors at baseline and outcome using univariate statistical tests. Those factors significant at the 5% level will be further considered as prognostic factors using a general linear model.

It is estimated that 780 subjects will provide more than 90% power to detect an effect size of less than 0.25 adjusted for other variables which have a co-efficient of determination (R-squared) with the outcome of up to 0.5. Assuming a 22% loss to follow up at 6 months, 1000 participants will initially be recruited.

**Discussion:**

This study may offer service users and providers with guidance to help identify whether or not physiotherapy is likely to be of benefit. Clinicians may have some direction as to what key factors indicate a patient’s likely response to physiotherapy.

## Background

Shoulder pain is a common and often persistent musculoskeletal problem affecting all ages, with a lifetime prevalence of one in three [1].

In 2011–2012, the prevalence of work related upper limb disorders in Great Britain exceeded that of low back pain [2]. Shoulder pain is one of the most common musculoskeletal disorders in the working population [3]. The cost of shoulder pain to healthcare and its effect on the economy is unclear. Both Dutch [4] and Swedish [5] studies have demonstrated that between 50 [4] and 84 [5] percent of overall costs due to shoulder pain is related to sick leave. Sickness absence due to shoulder pain in young working adults has been linked with high levels of sickness absence due to other diagnoses in subsequent years [6].

In primary care, shoulder pain is the third most common musculoskeletal presentation [7]. Of those people who chose to visit their General Practitioner with first episode shoulder pain, only 50 percent show complete recovery six months later [8]. At one year follow up, recovery rate increases by only 10 percent [8]. The most effective treatment for musculoskeletal shoulder pain is not yet known. Conservative management in the form of physiotherapy is therefore often recommended as the first choice of treatment [9]. Indeed, up to one third of patients referred for physiotherapy musculoskeletal outpatient services in primary care have shoulder pain [10]. Although physiotherapy constitutes a relatively low proportion of the overall cost, in relation to total healthcare costs, physiotherapy accounts for between 37 [4] and 60 percent [5].

When physiotherapy is unsuccessful, other interventions such as pain management clinics or surgery are often considered. However, there is already some evidence that longer duration of shoulder symptoms is associated with an increased likelihood of chronic pain [11]. It is therefore important, that wherever possible, the most appropriate treatment for any individual is identified early on in the clinical pathway.

At present, it is not possible to identify, from the initial physiotherapy assessment, which factors predict the outcome of physiotherapy for patients with shoulder pain. Research has shown that a range of biopsychosocial factors are related to outcome following surgical [12] or General Practitioner [11,13-15] management of shoulder pain. This study aims to identify which factors are related to outcome of shoulder pain following physiotherapy.

A greater knowledge of prognostic factors in terms of who is likely to respond to physiotherapy and who will not is vital for patients, healthcare professionals and commissioners and ensures effective and efficient use of limited resources. Additional benefits include:

●  Enabling patients to make an informed choice about whether or not they wish to pursue physiotherapy based on the prognosis of a positive outcome.

●  Facilitating greater confidence in physiotherapy treatment, leading to greater compliance and less non attendance.

●  Assisting physiotherapists in terms of effective and timely decision making and best utilization of limited resources.

●  Providing all health care professionals with a clinical reasoning tool to help distinguish between patients who will respond to physiotherapy, and those who will go on to have persistent symptoms and may benefit from earlier referral to the multidisciplinary team for a surgical opinion or chronic pain management.

●  Minimising the negative consequences of chronic pain and disability, for example associated depression, time off work, utilisation of health resources and disablement resettlement [16,17].

### Study objectives

The primary objective of this study is to identify which patient characteristics and baseline measures, commonly assessed at the first physiotherapy appointment, are related to the functional outcome of shoulder pain 6 weeks and 6 months after starting physiotherapy treatment.

The secondary objectives of this study are to:

i. describe a typical programme of physiotherapy for the management of shoulder pain and

ii. to describe the outcomes of physiotherapy in relation to physiotherapy treatments.

## Methods/Design

### Study design

A longitudinal cohort study of prognostic factors.

### Study setting

Potential participants will be selected from patients referred for the management of shoulder pain to participating physiotherapy out-patient musculoskeletal departments based in General Practice, primary and secondary care hospital sites in the Eastern Counties of England from November 2011 to September 2013.

### Eligibility criteria

Participants must be 18 years or older, with musculoskeletal shoulder pain of any duration, and reproduction of shoulder pain and/or restriction on active or passive movement in at least one direction. Participants must score 8 or more on the Shoulder Pain and Disability Index (SPADI) [18] or Quick Disability of the Arm, Shoulder and Hand Questionnaire (QuickDASH) [19].

In order to minimise confounding variables for recovery, participants presenting post operatively, with serious trauma or pathology to the shoulder, or with pathologies or syndromes which refer directly to the shoulder are excluded.

Exclusion criteria

●  Fracture of the affected shoulder in the last five years.

●  Dislocation of the affected shoulder which has resulted in a visit to secondary care and investigative radiology in the last five years.

●  Surgical intervention on the affected shoulder in the last five years. (This does not include hydrodilatation, manipulation under anaesthetic or local steroid injection).

●  Systemic condition with significant musculoskeletal component (i.e. inflammatory joint disease, polymyalgia rheumatica, neoplastic disorder).

●  Complex regional pain syndrome.

●  Predominant reproduction of shoulder pain on movements of the cervical or thoracic spine rather than shoulder movement.

●  Neck pain related to serious pathology, myelopathy or radiculopathy.

●  Known neoplasm.

### Care pathways and physiotherapy

The clinical pathway for patients recruited onto this observational study remains the same; physiotherapy treatment, referral pathways and waiting times are unaffected. Physiotherapy usually includes advice and exercise but may also include additional pain neuromodulatory techniques such as manual therapy or electrotherapy. The Chartered Society of Physiotherapy have developed evidence based guidelines for the management of some clinical presentations of shoulder pain [20,21]. Shoulder exercises have been demonstrated and recommended as an effective form of physiotherapy [20-23]. However as yet there is no evidence that one form of shoulder exercise is superior to another [22,23]. Physiotherapy guidelines and reviews generally recommend passive pain modulatory treatments as adjuncts to exercise rather than stand alone treatments [20,21,23-25]. With the exception of exercise, there is currently no convincing evidence that any one type of physiotherapy treatment is superior to another [9,23].

### Prognostic factors

The International Classification of Functioning, Disability, and Health [26] highlight the importance of an integrative approach to patient assessment in which impairment, activity, and participation are considered. The potential prognostic factors recorded at the initial physiotherapy appointment for this study will reflect this. Potential prognostic variables will include:

●  Demographic details.

●  Individual participant characteristics.

●  Lifestyle, psychosocial factors, past experience and expectations of physiotherapy.

●  Shoulder symptoms and general health.

●  Signs of impairment from the objective/clinical examination.

●  Activity limitations and participation restrictions.

### Outcome measures

The primary outcome measure is the Shoulder Pain and Disability Index (SPADI) [18,27]. This region specific self report questionnaire is designed to measure pain and disability associated with shoulder pain of musculoskeletal or undetermined origin. Thirteen items, covering two domains (pain and disability) are scored on a numerical rating scale between zero (no pain/difficulty) and ten (worst pain imaginable/so difficult it requires help). Each domain carries equal weighting in the overall score which is expressed as a percentage where zero represents no pain or disability and 100% represents maximum pain and disability. Previous research has demonstrated no floor and no or very low ceiling effects, excellent reliability [28], responsiveness [29] and ability to discriminate between different severities of shoulder pain [30].

The secondary outcome of the study is the Quick Disability of the Arm, Shoulder and Hand Questionnaire (QuickDASH) [31]. The QuickDASH is a broader region specific patient rated questionnaire. Eleven items, covering six domains (daily activities, symptoms, social function, work function, sleep, and confidence) are scored on a numerical rating scale between one (no difficulty) and five (unable). The overall score is expressed as a percentage where zero represents no disability and 100% represents maximum disability. Previous research has demonstrated good responsiveness [31,32] and similar validity, reliability and precision to the Disability of the Arm, Shoulder and Hand Questionnaire [33].

### Sample size

A formal sample size calculation is difficult given the lack of published information on correlations between putative prognostic factors and outcome. However, using the approach suggested by Lipsitz and Parzen [34] 780 subjects would provide more than 90% power to detect an effect size of less than 0.25 adjusted for other variables which have a co-efficient of determination (R-squared) with the outcome of up to 0.5. Assuming a 22% loss to follow up at 6 months, 1000 participants will initially be recruited.

An initial survey of six of the participating sites over the period March 2009–2010 indicated that 1500 potential participants with shoulder pain were referred over a 12 month period. However changes in working practice and referral patterns, and the addition of more exclusion criteria indicate that further sites will be required to meet this target. Additional NHS healthcare trusts/social enterprises, some of which include a number of potential locations, have therefore been included as potential sites with some of these contingency sites. Rates of recruitment and completion rates at 6/52 and 6/12 will be monitored each month and additional sites recruited as indicated.

### Participant selection and recruitment

**Figure 1:**
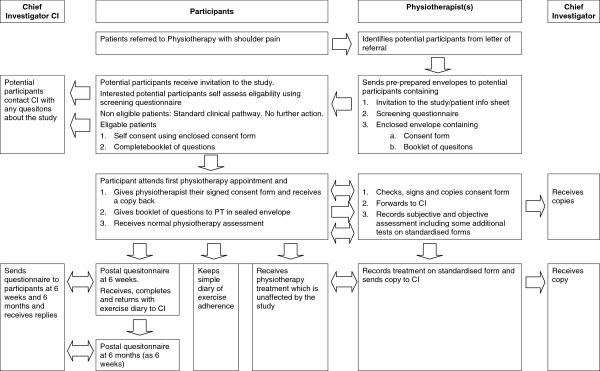
Patient pathway with reference to physiotherapy and the chief investigator.

Potential participants will be identified at each site from the letter of referral. At a similar time to the first physiotherapy appointment being posted to the patient, a separate envelope containing an invitation pack will be sent to all potential participants. See Figure 1 for an outline of the patient pathway. The invitation pack, previously prepared, stamped and signed by the Chief Investigator will be non-personalised and will contain a Patient Information Sheet. This will clearly state that involvement in the study is voluntary and that participants will be free to withdraw from the study at any time. It will also include contact details of the Chief Investigator to provide the opportunity for further questions. A variety of contact methods will be provided; e-mail, study mobile number, landline with 24 hour answer machine and postal address.

A self screening questionnaire, key questions of which are presented in Additional file 1, will be provided within the invitation pack. If the patient wishes to take part in the study they will be asked to complete the screening questionnaire at home prior to their first physiotherapy appointment.

### Informed self consent

If the patient appears eligible upon self screening and wishes to take part in the study they will also be asked to complete the consent form enclosed within the invitation pack. On arriving for their first physiotherapy appointment, potential participants who fulfill the eligibility criteria via the screening questionnaire and provide written informed consent will be enrolled onto the study. The physiotherapist treating the participant will check the screening and consent forms have been accurately completed.

The lead physiotherapist at each site will be asked to keep a log of the number of patients who are eligible and consent versus those who are eligible and do not consent. In addition the age, sex and index of social deprivation of all potential participants will be anonymously recorded to allow comparisons between consenters and non consenters.

### Collection of baseline prognostic factors

Predetermined factors, which may predict outcome from physiotherapy will be measured and collected prior to and during the first physiotherapy appointment via three methods; the participant will complete a “booklet of questions” provided within their patient invitation pack and their physiotherapist will record selected details of their initial subjective and clinical assessment on a clinical record form.

#### Demographic and individual participant characteristics including activity and participation

Data about demographics and individual characteristics will be collected via an eight page A4 sized “booklet of questions” which will be completed by the patient at home prior to their first physiotherapy appointment. This contains individual questions about the participant and the characteristics of their shoulder pain, their work, everyday activities, and lifestyle, their experience of physiotherapy in the past, motivational factors and expectations of physiotherapy for their current shoulder symptoms. In addition the booklet contains four validated self report questionnaires. These have been selected based on their psychometric properties in similar populations and respondent burden. The Pain Self Efficacy Scale [35] requires the participant to state their confidence, despite their pain, on a scale of 0–6 in 10 domains. The total score ranges from 0–60 where a higher score represents greater self efficacy beliefs. The “Godin leisure time exercise questionnaire” [36-38] requires participants to state how often they perform mild, moderate and strenuos exercise per week and how often they “work up a sweat”. The results are used to calculate weekly metabolic equivalents (METs). Pain, symptoms and disability will be measured using the SPADI [18] and QuickDASH [31]. The participant will also be asked to estimate their expected recovery on a seven point global impression of change. Due to the sensitive nature of some of the topics participants will be asked to place the “booklet of questions” in a sealed envelope before giving it to their physiotherapist at their first appointment.

#### Shoulder symptoms and general health

Details about shoulder symptoms and general health will be collected and recorded in part by the participant within the “booklet of questions” and in part by the physiotherapist during the subjective assessment. Physiotherapists will record details of co-morbidities, work status and the distribution, cause, onset and duration of the participant’s current shoulder symptoms on a clinical record form specifically designed for this study.

#### Signs of impairment from the objective/clinical examination

Signs of impairment will be measured and recorded by the participant’s physiotherapist during the objective/physical examination on the clinical record form. This will include the following: the effect of spinal movement on shoulder pain, the range and limiting factor for active and passive flexion, abduction and external rotation of the shoulder, myometry readings for painfree isometric abduction and external rotation, response to manual facilitation of the scapula during shoulder elevation, and the physiotherapist’s assessment of the clinical problem. Finally, based on their assessment, physiotherapists will provide an estimate of the participant’s predicted recovery during the course of physiotherapy on a seven point global impression of change. All participating physiotherapy departments will receive a formal site initiation visit by the Chief Investigator during which training on standard operating procedures for data collection will take place. Physiotherapists will be also be provided with written definitions and quick reference illustrations of standardised operating procedures for each clinical examination used in the study.

### Collection of outcome measures

Outcome measures (SPADI, QuickDASH and Global Impression of Change) will be collected by postal self report questionnaires 6 weeks and 6 months after commencing physiotherapy. The participant will not have access to the responses they provided to these same questions at baseline or at previous follow ups. The chief investigator will be blinded to previous data entries when inputting data from the 6 week and 6 month follow up questionnaires. To maximise response rates, non-responders will be sent two text messages, e-mails or postal reminders to return their questionnaires. The “Booklet of Questions” filled in prior to the first physiotherapy appointment will ask each participant which of these methods the participant prefers.

### Collection of details of physiotherapy treatment, attendance and exercise adherence

Physiotherapists will record details of treatment and the participant’s attendance/non attendance, on a customised clinical record form specifically designed for this study. This will be completed and then returned to the Chief investigator when the participant is discharged from physiotherapy. Participants will record exercise adherence for the first six weeks after their initial physiotherapy appointment using a simple diary specifically designed for the study and provided by the physiotherapist at their initial assessment. This will be returned to the Chief Investigator with the 6 week follow up questionnaire. Specific details of exercise adherence over the longer term will be requested within the 6 month follow up questionnaire.

### Collection of details of treatment other than physiotherapy

Details of any treatments received in addition to that provided by the participant’s NHS physiotherapist (including increase in pain medication) will be requested within the 6 week and 6 month follow up questionnaire.

### Adverse events

It is not expected that any adverse events will occur as a result of this observational study. However Principal Investigators at each site will be asked to record and notify the Chief Investigator should any occur. Information about participants who worsen with physiotherapy will be captured at 6 week and 6 month follow up.

### Data management

Data collected at each site will be sent to the Chief Investigator via internal post or via registered courier. Data will then be transferred using a Microsoft access data base, onto a password protected file store. Handling of all personal data from patients who have consented to be in the study will be done in compliance with the Data Protection Act [39]. The dataset held will be pseudoanonymised meaning that a unique identifier will be used to link patients’ data to their personal details. Personal details are required by the Chief Investigator to send questionnaires to participants’ home addresses at 6 week and 6 month follow up.

Data values will be electronically range checked during entry onto the access database. Unusual or unexpected clinical findings at baseline will be discussed with the participant’s physiotherapist for further clarity. Physiotherapists and participant’s will be contacted for missing data. All anonymised data entries will be double checked for accuracy and any amendments recorded.

Anonymised data will be transferred to STATA (Timberlake UK) for statistical analysis.

### Statistical analysis

An initial exploratory analysis will be used to assess the relationship between potential prognostic factors at baseline and outcomes at the two follow-up time points using univariate statistical tests. Those factors that are significant at the 5% level will be further considered as prognostic factors using a general linear model. A stepwise selection process, based on change in scaled deviance, will be used for model construction. Residuals will be examined to assess the assumption of a Normal distribution. In the case that a Normal distribution cannot be assumed, transformations, such as a logarithmic transformation will be used. Sensitivity analyses will be carried out by removing extreme and influential observations and assessing changes on the estimates of the model parameters.

Data on physiotherapy treatment will be presented in a descriptive manner. An exploratory analysis of the potential correlation between treatment variables and outcome will be conducted although the former will not be considered as prognostic factors as they are clearly not available to patients or health professionals prior to the commencement of physiotherapy.

Baseline measurements will be compared between participants who complete and those who do not complete data collection at 6 week and 6 month follow up periods to assess the potential to bias the results.

### Patient and public involvement

The preliminary work undertaken for this proposal has been in collaboration with the local Public and Patient Involvement in Research (PPIRes) project who are linked to INVOLVE, the national advisory group, funded by the National Institute for Health Research (NIHR). The group has provided feedback on previous draft proposals on research for Physiotherapy and shoulder pain and agreed that this was an important area for research. Recruitment and procedural suggestions from the consumers’ perspective were offered during the development of the protocol. The project steering group includes two members of PPIRes who will provide on-going feedback on revisions of participant documentation and suggestions to maximise participant recruitment. PPIRes members of the steering group will play a key role in dissemination of the results to the public.

### Service provider involvement

Principal Investigators whose sites would definitely be involved in recruiting participants attended two four hour workshops at the University of East Anglia in April 2011 and October 2011. The initial workshop focused on procedural details, in particular upon reaching a consensus of which prognostic factors it would be feasible to collect, by whom and by what methods and measurements. This necessitated achieving a balance between the limited evidence base, expert opinion and operator burden (physiotherapist and potential participant). The group also discussed recruitment and consenting procedures and methods for recording treatment. The second workshop focused upon good clinical practice requirements and applications to the study, the content of site initiation visits, and problem solving for potential recruitment and consenting issues.

## Discussion

Shoulder pain affects basic activities of daily living such as toileting and dressing, many recreational and sporting activities and is one of the most common musculoskeletal conditions in the working population. A lack of appropriate and timely intervention can lead to social exclusion, lost economic productivity in terms of sickness absence and worklessness. This obviously has an impact on the prosperity and economic recovery of any nation.

Increasingly demands on health services for an ever-growing population mean that difficult choices are being made in terms of where resources are best allocated for an effective quality service. Quality care demands that treatment provided is tailored to provide the most effective care pathway for any given individual.

Everyday decisions made by clinicians have an impact on the quality of care provided and experienced by patients [40]. Prompt selection of the appropriate clinical pathway for individuals with musculoskeletal shoulder pain is essential – some but not all patients with shoulder pain will respond to physiotherapy. Inappropriate referral down a less effective route and/or delayed referral to more appropriate care are more likely to lead to long-term chronic or persistent pain. Chronic pain is less likely to respond to treatment. In addition to the personal burden for the individual, continued referral from one department to another is a costly, ineffective, and inefficient use of health resources.

This study will provide service users and providers with some of the information necessary to identify whether or not physiotherapy is likely to be of benefit. For the first time clinicians will have some guidance as to what key factors indicate a patients likely response to physiotherapy. Physiotherapy is a relatively safe, non-invasive, and for many, an effective intervention. This valuable resource can therefore be utilized to maximum effect. Individuals who are unlikely to respond to physiotherapy can be promptly referred to other services so increasing the likelihood of their success.

This study will provide a basis for future research to develop and validate a clinical prediction rule for physiotherapy and shoulder pain. It will also help to describe what constitutes “standard physiotherapy” for musculoskeletal shoulder pain in England, and allow a more rigorous justification of the “control” arm of physiotherapy when comparing the effectiveness of other physiotherapy treatments or treatments other than standard physiotherapy.

### Ethical approval

The NRES Committee East of England – Norfolk provided ethical approval for this study in June 2011. Rec Reference number 11/EE/0212, Protocol number R18870.

## Competing interests

The authors declare that they have no competing interests

## Authors’ contributions

RC conceived of the study and drafted the original manuscript. All authors contributed to the development and critical evaluation of the study protocol. All authors were involved in revisions and final approval of the manuscript.

## Authors’ information

The Chief Investigator (RC) for this study is funded by a Clinical Doctoral Research Fellowship from the National Institute for Health Research (NIHR CAT CDRF 10–008).

LS and CJH are academic supervisors and JS the clinical supervisor. The views and opinions expressed within this protocol are those of the authors and do not necessarily reflect those of the NIHR, NHS or the Department of Health.

## Pre-publication history

The pre-publication history for this paper can be accessed here:

http://www.biomedcentral.com/1471-2474/14/192/prepub

## Supplementary Material

Additional file 1 Questions on the self-screening questionnaire. Click here for file
