# Supplementary material for: Predicting response to physiotherapy treatment for musculoskeletal shoulder pain: protocol for a longitudinal cohort study
Source: BMC Musculoskelet Disord. 2013 Jun 21;14:192. doi: 10.1186/1471-2474-14-192 (PMC3720561; doi:10.1186/1471-2474-14-192)
Supplement: Additional file 1 — Questions on the self-screening questionnaire. [file 1471-2474-14-192-S1.pdf]

Additional File 1: Questions on the self-screening questionnaire.

1. Have you been referred to physiotherapy for a problem with your shoulder?	Yes ☐	No ☐
2. Do your shoulder symptoms get worse, even for just a moment or two, when you move or place your arm in a particular position?	Yes ☐	No ☐
3. Have you had an operation on your shoulder in the last five years?	Yes ☐	No ☐
4. Have you broken or fractured one of the bones in your shoulder in the last five years?	Yes ☐	No ☐
5. Has your shoulder "popped out of joint" or dislocated in the last five years?	Yes ☐	No ☐
6. Do your shoulder symptoms get worse when you move or place *your neck* rather than your shoulder in a particular position?	Yes ☐	No ☐
If all your answers fall in the grey shaded boxes, you have the type of shoulder symptoms we are investigating in our study.		
